# Disarming the Host: Detoxification of Plant Defense Compounds During Fungal Necrotrophy

**DOI:** 10.3389/fpls.2021.651716

**Published:** 2021-04-30

**Authors:** Nathaniel M. Westrick, Damon L. Smith, Mehdi Kabbage

**Affiliations:** Department of Plant Pathology, University of Wisconsin–Madison, Madison, WI, United States

**Keywords:** detoxification, necrotrophy, fungal pathogen, phytoalexin, phytoanticipin

## Abstract

While fungal biotrophs are dependent on successfully suppressing/subverting host defenses during their interaction with live cells, necrotrophs, due to their lifestyle are often confronted with a suite of toxic metabolites. These include an assortment of plant defense compounds (PDCs) which can demonstrate broad antifungal activity. These PDCs can be either constitutively present in plant tissue or induced in response to infection, but are nevertheless an important obstacle which needs to be overcome for successful pathogenesis. Fungal necrotrophs have developed a number of strategies to achieve this goal, from the direct detoxification of these compounds through enzymatic catalysis and modification, to the active transport of various PDCs to achieve toxin sequestration and efflux. Studies have shown across multiple pathogens that the efficient detoxification of host PDCs is both critical for successful infection and often a determinant factor in pathogen host range. Here, we provide a broad and comparative overview of the various mechanisms for PDC detoxification which have been identified in both fungal necrotrophs and fungal pathogens which depend on detoxification during a necrotrophic phase of infection. Furthermore, the effect that these mechanisms have on fungal host range, metabolism, and disease control will be discussed.

## Introduction

To successfully infect and colonize a host, phytopathogenic fungi must overcome a repertoire of plant defense compounds (PDCs) with broad antimicrobial properties. PDCs are often sub-classified as those which are induced in response to infection (phytoalexins) and/or those that exist as pre-formed antimicrobial compounds in plant tissue (phytoanticipins). These classifications are not mutually exclusive, as compounds may exist as both a phytoalexin and phytoanticipin within a single plant (Van Etten et al., 1994). The lifestyle of a pathogen can play a major role in how they interact with these compounds, as phytoalexin production is often dependent on the successful induction of plant defenses and phytoanticipin activity requires damage to plant tissue (Tiku, 2020). In the case of biotrophic pathogens that depend on the colonization of living tissue, it has been suggested that the maintenance of plant cellular integrity may prevent the release of phytoanticipins, thus subverting their role in plant defense (Glenn et al., 2002). Additionally, pathogens with greater host-specialization, as is seen in nearly all biotrophs, tend to present greater basal tolerance for co-evolved host antimicrobials and may be less dependent on specific detoxification mechanisms (Basse, 2005; Buxdorf et al., 2013). This may be why active detoxification of host PDCs by fungal biotrophs has only been noted in rare circumstances (Okmen et al., 2013).

Contrarily, necrotrophic and hemibiotrophic fungi, which induce cell death and compromise the integrity of plant tissues during colonization, do not benefit from this covertness and must actively detoxify host antimicrobials. This detoxification is facilitated by a number of mechanisms including metabolization of the compounds to less toxic derivatives and transporter-mediated efflux to maintain PDCs at sublethal thresholds. This review will serve to summarize the current state of knowledge surrounding the detoxification of plant PDCs during fungal necrotrophy and the implications this detoxification has on pathogen evolution, host range, and management.

## Enzymatic Detoxification of Inducible PDCs

Inducible PDCs are defined in this review as low molecular weight antimicrobial compounds that can be produced/accumulated in response to pathogen invasion. This category includes all compounds classified as phytoalexins (i.e., induced in response to infection) and those which are both phytoalexins and phytoanticipins (i.e., compounds which exist in healthy plant tissue and additionally accumulate in response to infection). Examples of such compounds include the antimicrobials resveratrol and maackiain (Van Etten et al., 1994; Wang et al., 2013). While these compounds are often broadly mycotoxic, it has been observed for decades that pathogens of specific plants appear more tolerant of their PDC repertoire than non-pathogens (Delserone et al., 1999). This tolerance is a necessary trait of a pathogen that seeks to fully colonize host tissue and, in many cases, appears to result from its capacity to actively metabolize (detoxify) specific PDCs into less toxic derivatives (Table 1). Similar to the production of phytoalexins by the plant, this detoxification is typically inducible through fungal exposure to mycotoxic compounds or in response to specific cues (George and Van Etten, 2001; Pedras et al., 2004; Pedras et al., 2007; Chen et al., 2019). This stepwise induction follows: (1) pathogen recognition by the plant, (2) production and accumulation of PDCs at and around the site of infection, (3) recognition of PDCs by the pathogen, (4) production of detoxification enzymes. Some PDCs are not only antimicrobial themselves, but act as intermediates in the biosynthesis of other, often more mycotoxic compounds (Pedras et al., 2008; Dubrovina and Kiselev, 2017). Because of this, the metabolism of such compounds, including resveratrol and brassinin (discussed below), can serve to both actively protect the fungus and disrupt the production of other bioactive compounds. The following section discusses the enzymatic detoxification of inducible PDCs that have been observed or characterized in fungal necrotrophic/hemibiotrophic pathogens of plants.

**TABLE 1:** Metabolization of inducible PDCs by fungal necrotrophs and hemibiotrophs.

Class	Compound	Fungal species	Conversion product	GEC	Citation
**Pterocarpans**	Pisatin	*N. haematococca*	(+)-6a-Hydroxymaackiain	+	Delserone et al., 1999
		*Stemphylium botryosum*	(+)-6a-Hydroxymaackiain	−	Higgins, 1981
		*F.oxysporum* f. sp.*pisi*	6a-Hydroxy-inerminisoflavan	+	Coleman et al., 2011a
	Maackiain	*S.botryosum*	Dihydromaackiain	−	Pedras and Ahiahonu, 2005
		*F. solani*	la-Hydroxymaackiain 6a-Hydroxymaackiain	+ +	Covert et al., 1996 Covert et al., 1996
		*S. trifoliorum*	6a-Hydroxymaackiain	−	Macfoy and Smith, 1979
		*B. cinerea*	6a-Hydroxymaackiain	−	Macfoy and Smith, 1979
	Medicarpin	*F. solani*	la-Hydroxymaackiain 6a-Hydroxymedicarpin	+ +	Denny and Van Etten, 1982 Denny and Van Etten, 1982
		*F. proliferatum*	3,9-Dihydroxypterocarpan	−	Pedras and Ahiahonu, 2005
		*S. botryosum*	Vestitone	−	Pedras and Ahiahonu, 2005
		*S. trifoliorum*	Vestitol	−	Pedras and Ahiahonu, 2005
		*B. cinerea*	6a-Hydroxy derivative	−	Pedras and Ahiahonu, 2005
		*Colletotrichum lindemuthianum*	6a-Hydroxy derivative	−	Ingham, 1976
		*Colletotrichum coffeanum*	6a-Hydroxy derivative	−	Ingham, 1976
		*Ascochytarabiei*	Multiple pterocarpan derivatives	−	Kraft et al., 1987
	Phaseollidin	*F. solani*	Phaseollidin hydrate	+	Turbek et al., 1992
	Phaseollin	*S.botryosum*	Phaseollinisoflavan	−	Higgins et al., 1973
		*C.lindemuthianum*	6*a*-Hydroxyphaseollin 6a7-Dihydroxyphaseollin	− −	Pedras and Ahiahonu, 2005 Pedras and Ahiahonu, 2005
		*F. solani* f. sp. *phaseoli*	1*a*-Hydroxy phaseollone	−	Pedras and Ahiahonu, 2005
		*Phaeosphaeria nodorum*	*cis* and *trans* 12,13-dihydrodihydroxyphaseollin.	−	Pedras and Ahiahonu, 2005

**Isoflavones**	Kievitone	*F. solani*	Kievitone hydrate	+	Daoxin et al., 1995
	2,3-Dehydrokievitone	*Aspergillus flavus*	Dihydrofurano-isoflavone Dihydropyrano-isoflavone 2,3-Dehydrokievitone glycol	− − −	Pedras and Ahiahonu, 2005 Pedras and Ahiahonu, 2005 Pedras and Ahiahonu, 2005
		*B. cinerea*	Dihydrofurano-isoflavone Dihydropyrano-isoflavone 2,3-Dehydrokievitone glycol	− − −	Pedras and Ahiahonu, 2005 Pedras and Ahiahonu, 2005 Pedras and Ahiahonu, 2005
	Formononetin	*F. avenaceum*	Calycosin	−	Pedras and Ahiahonu, 2005
	Biochanin A	*F.oxysporum* f. sp.*lini (*and *lycopersici*)	Pratensein	−	Weltring et al., 1982
	Daidzein	*Aspergillussaitoi*	8-Hydroxydaidzein	−	Esaki et al., 1998
	Genistein	*A. saitoi*	8-Hydroxygenistein	−	Esaki et al., 1998
		*Armillaria mellea*	4-Hydroxyphenylacetic 1,3,5-Trihydroxybenzene	− −	Curir et al., 2006 Curir et al., 2006

**Other flavonoidsand stilbenoids**	Resveratrol	*B. cinerea*	Resveratrol *trans*-dehydrodimer	+	Schouten et al., 2002
	Quercetin	*S. sclerotiorum*	2-PCPGCA	+	Chen et al., 2019
		*P. olsonii*	Unknown product	+	Tranchimand et al., 2008
		*Verticillium dahliae*	2-PCPGCA	+	El Hadrami et al., 2015
	Kaempferol	*P. olsonii*	Unknown product	+	Tranchimand et al., 2008
		*S. sclerotiorum*	2,4-Dihydroxy-6-[(4-hydroxybenzoyl)oxy] benzoic acid	+	Chen et al., 2019
	Galangin	*P. olsonii*	Unknown product	+	Tranchimand et al., 2008
	Fisetin	*P. olsonii*	Unknown product	+	Tranchimand et al., 2008
	Rutin	*V. dahliae*	Quercetin	−	El Hadrami et al., 2011
		*flavus*	Protocatechuic acid 2-PCPGCA Rutinose	−	Westlake et al., 1961
	Sakuranetin	*Rhizoctonia solani*	Sakuranetin-4’-O-β-D-xylopyranoside Naringenin-7-O-β-D-xylopyranoside	− −	Katsumata et al., 2018 Katsumata et al., 2018
		*M. oryzae*	Naringen Sternbin	−	Katsumata et al., 2017
	Astringin	*Endoconidiophora polonica*	Muconoid-typederivatives	+	Wadke et al., 2016
	Catechin	*E.polonica*	Muconoid-type derivatives	+	Wadke et al., 2016

**Indoles**	Brassinin	*Leptosphaeria maculans*	3-Indolecarboxaldehyde 3-Indolecarboxylic acid Indolyl-3-methanamine	+ + +	Pedras et al., 2008 Pedras et al., 2007 Pedras et al., 2007
		*Alternaria brassicicola*	Indolyl-3-methanamine	+	Pedras et al., 2009
		*S. sclerotiorum*	1-b-D-glucopyranosyl (b-D-glc) brassinin	+	Sexton et al., 2009
	1-Methoxybrassinin	*S. sclerotiorum*	Spirothiazolidinone Spirothiazolidinethione	− −	Pedras and Hossain, 2006 Pedras and Hossain, 2006
			7-(b-D-glc)-1- methoxybrassinin	−	Pedras and Ahiahonu, 2005
	Cyclobrassinin	*S. sclerotiorum*	1-(b-D-glc) cyclobrassinin	−	Pedras and Hossain, 2006
		*R. solani*	5-Hydroxybrassicanal A	−	Pedras and Ahiahonu, 2005
		*L.maculans*	dioxibrassinin Brassilexin	− −	Pedras and Ahiahonu, 2005 Pedras and Ahiahonu, 2005
	Brassilexin	*S. sclerotiorum*	1-(b-D-glc) brassilexin	−	Pedras and Hossain, 2006
	Spirobrassinin	*S. sclerotiorum*	Spirooxathiazolidinone	−	Pedras and Hossain, 2006
	Brassicanal A	*L.maculans*	3-Methylindolyl-2-methylsulphoxide	−	Pedras and Ahiahonu, 2005
		*S. sclerotiorum*	1-(b-D-glc) brassicanal A	−	Pedras and Hossain, 2006
	Camalexin	*R. solani*	5-hydroxycamalexin	−	Pedras and Ahiahonu, 2005
		*S. sclerotiorum*	1-(b-D-glc) camalexin	−	Pedras and Hossain, 2006
		*B. cinerea*	Indolethiocarboxamide	−	Pedras et al., 2011
	6-Methoxy camalexin	*S. sclerotiorum*	1-(b-D-glc) camalexin	−	Pedras and Hossain, 2006

**Terpenoids**	Capsidiol	*B. cinerea*	Capsenone	−	Pedras and Ahiahonu, 2005
		*F. oxysporum* f. sp. *vasinfectum*	Capsenone	−	Pedras and Ahiahonu, 2005
	Lubimin	*Gibberella pulicaris*	2-Dehydrolubimin	−	Pedras and Ahiahonu, 2005
		*Penicillium chrysogenum*	15-Dihydrolubimin	−	Pedras and Ahiahonu, 2005
	3-Hydroxylubimin	*P. chrysogenum*	3-Hydroxy-l5-dihydrolubimin	−	Pedras and Ahiahonu, 2005
	Rishitin	*G.pulicaris*	13-Hydroxyrishitin 11,12-Epoxyrishitin	− −	Pedras and Ahiahonu, 2005 Pedras and Ahiahonu, 2005
	Linalool	*B. cinerea*	Multiple monoterpenes	−	Shimizu et al., 1982
	Limonene	*Grosmannia clavigera*	Limonene-1,2-diol	+	Wang et al., 2014
	Momilactone A	*M. oryzae*	3,6-Dioxo-19-nor9β-pimara-7,15-diene	−	Imai et al., 2012
	Monoterpenes	*Heterobasidion parviporum*	Multiple derivatives	−	Kusumoto et al., 2014

**QN**	o-Hibiscanone	*V.dahliae*	Hydroquinone derivative	−	Pedras and Ahiahonu, 2005

**FFA**	Wyerone epoxide	*B. cinerea* *B. fabae*	Wyerole poxide Dihydrodihydroxywyerol	−	Pedras and Ahiahonu, 2005 Pedras and Ahiahonu, 2005

*GEC,gene or enzyme characterized?; QN,quinone; FFA,furanoid fatty acids.*

### Pterocarpans

**FIGURE 1:**
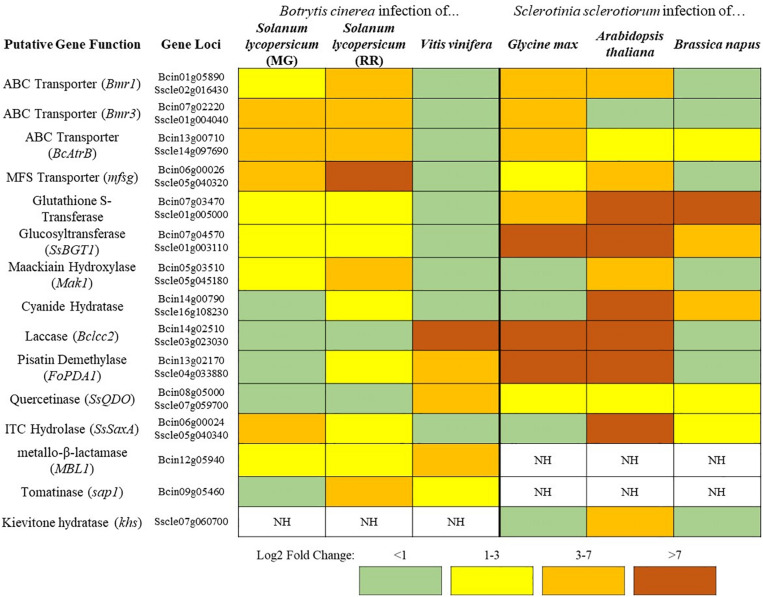
A sampling of *Botrytis cinerea* and *Sclerotinia sclerotiorum* genes putatively involved in detoxification of PDCs. Simplified differential gene expression values are presented for *B. cinerea* [infecting mature green (MG) and red ripe (RR) *Solanum lycopersicum* and *Vitis vinifera*] and *S. sclerotiorum* (infecting *Glycine max, Arabidopsis thaliana*, and *Brassica napus*) (Haile et al., 2017; Seifbarghi et al., 2017; Petrasch et al., 2019; Peyraud et al., 2019; Westrick et al., 2019). All samples were harvested at ∼24 h post inoculation and differential expression values were generated through comparison to *in vitro* culture controls. NH = no homolog found in the genome.

Pterocarpans are class of inducible PDC produced in the phenylpropanoid pathway that are primarily associated with legume defense against biotic stress (Dixon et al., 2002). Pisatin is an antifungal pterocarpan that is produced in pea pods of *Pisum sativum* during pathogen challenge (Perrin and Bottomley, 1961). As one of the earliest discovered PDCs, pisatin was initially credited with driving non-host resistance in *P. sativum* to fungal pathogens, as only fungi adapted to this host environment are able to cause disease (Delserone et al., 1999). Pisatin detoxification in *Nectria haematococca* was attributed to microsomal cytochrome P450s capable of efficiently demethylating pisatin to the less toxic (+)-6a-hydroxymaackiain, and termed pisatin demethylases (PDAs) (George et al., 1998; George and Van Etten, 2001). The gene encoding the primary PDA (*PDA1*) is located, along with several other pea pathogenicity genes (PEP genes), on a 1.6 Mb supernumerary chromosome which is dispensable for fungal survival in culture, but critical for pathogenicity on pea (Miao et al., 1991; Han et al., 2001). While the function of most of these PEP genes is still unknown a single gene, PEP5, was found to be a likely efflux transporter (discussed in section “Toxin Efflux”), capable of increasing virulence on pea in the absence of other detoxification mechanisms (Han et al., 2001). Although PDA genes have been primarily characterized in *Fusarium* spp., PDA activity has been noted in other fungal pathogens of pea and putative orthologs are induced during infection in some broad host range necrotrophs (Table 1 and Figure 1) (George and Van Etten, 2001).

A closely related pterocarpan, maackiain, plays a similar role to pisatin in chickpea defense and its detoxification is also highly correlated with *N. haematococca* virulence (Lucy et al., 1988). Three loci involved in the degradation of maackiain, and the related medicarpin, have been identified (MAK1-3), but only *MAK1* has been fully characterized as a hydroxylase capable of converting maackiain into 1α-hydroxy-maackiain (Miao and Van Etten, 1992; Enkerli et al., 1998). Similar to *PDA1*, *MAK1*, and *MAK2* are on dispensable chromosomes associated with other putative virulence genes and *MAK1* appears to share a chromosome with a putative PDA gene (*PDA6-1*) thought to be involved in pisatin tolerance (Covert et al., 1996). Disruption mutants of *MAK1* have a reduced virulence and maackiain tolerant strains overexpressing *MAK1* demonstrate increased aggression, confirming a previously noted correlation between maackiain detoxification and virulence (Enkerli et al., 1998). While it is assumed that a tolerance for maackiain is a prerequisite for pathogenicity on chickpea and the degradation of this pterocarpan has been observed in other chickpea pathogens, no other genes involved in maackiain degradation have been characterized (Birgit et al., 1989; Tenhaken et al., 1991).

Another pterocarpan, phaseollidin, is known to accumulate during infection of the common bean, *Phaseolus vulgaris*, along with the isoflavonol kievitone (discussed below), and is degraded by some pathogens of *P. vulgaris* (Van Etten et al., 1989; Turbek et al., 1992). Early evaluation of phaseollidin metabolism by the pathogen *Fusarium solani* suggested that a single enzyme was detoxifying both compounds through a hydration reaction (Kuhn and Smith, 1979). Later work determined that distinct proteins are responsible for kievitone and phaseollidin hydratase (PHase) activities, although the gene encoding the latter has never been characterized (Turbek et al., 1992). As phaseollidin is the precursor of other antifungal PDCs including phaseollin, PHase activity may serve to both detoxify an important PDC and suppress the formation of other antimicrobials (Dewick and Steele, 1982). This may also explain why PHase activity is accomplished by a secreted extracellular enzyme, while other pterocarpan detoxifying proteins are intracellular (Table 2).

**TABLE 2:** Characterized genes encoding detoxification enzymes from fungal pathogens.

Species/substrate	Gene name	Protein type	Putative localization	Accession #
**Isoflavones**				
*F. solani*	*khs*	Kievitone Hydratase	Extracellular	L39639
*N. haematococca*	*NhKHS*	Kievitone Hydratase	Extracellular	XM_003041138
**Pterocarpans**				
*F. oxysporum*	*FoPDA1*	Pisatin Demethylase (CYP450)	Microsome	39939259
*N. haematococca*	*PDA1*	Pisatin Demethylase (CYP450)	Microsome	XM_003044179
*N. haematococca*	*MAK1*	Maackiain hydroxylase	Peroxisome	U35892
*F. solani*	*-*	Phaseollidin hydratase	Extracellular*	−
**Flavonols**				
*P. olsonii*	*poquer1*	Quercetinase	Extracellular	EU126643
*S. sclerotiorum*	*SsQDO*	Quercetinase	Extracellular	XM_001587370
*V. dahliae*	*VdQase*	Quercetinase	Cytoplasmic	XM_009653185
**Terpenoids**				
*G. clavigera*	CMQ_7007	Baeyer–Villiger monooxygenase	Peroxisome	XP_014176168
*G. clavigera*	CMQ_6956	Baeyer–Villiger monooxygenase	Peroxisome	XP_014176117
**Stilbenoids**				
*B. Cinerea*	*BcLcc2*	Laccase	Extracellular	XM_001553136
*E. polonica*	*EpCDO1*	Catechol dioxygenase	Cytoplasmic	KU221039
*E. polonica*	*EpCDO2*	Catechol dioxygenase	Cytoplasmic	KU221040
*E. polonica*	*EpCDO3*	Catechol dioxygenase	Cytoplasmic	KU221041
*E. polonica*	*EpCDO4*	Catechol dioxygenase	Extracellular	KU221042
**Indoles**				
*S. sclerotiorum*	*SsBGT1*	Glucosyltransferase	Extracellular	XM_001589312
**Hydrogen cyanide**				
*G. sorghi*	*Cht*	Cyanide hydratase	Cytoplasmic	M99044
*L. maculans*	*Cht*	Cyanide hydratase	Cytoplasmic	AF192405
**Benzoxazinoids**				
*F. verticillioides*	*Fdb1 (Mbl1)*	Metallo-β-lactamase (MBL)	Cytoplasmic	XM_018897183
*F. pseudograminearum*	*Fdb1 (Mbl1)*	MBL	Cytoplasmic	XM_009261242
*F. graminearum*	*Fdb1 (Mbl1)*	MBL	Cytoplasmic	VTO91902
*F. verticillioides*	*Fdb2 (Nat1)*	Arylamine *N*-acetyltransferase (NAT)	Cytoplasmic	XM_018901986
*F. pseudograminearum*	*Fdb2 (Nat1)*	NAT	Cytoplasmic	XM_009261241
*F. graminearum*	*Fdb2 (Nat1)*	NAT	Cytoplasmic	VTO91902
**Saponins**				
*G. graminis*	*Avn*	Avenacinase	Extracellular	U35463
*S. lycopersici*	*Tom*	Tomatinase	Extracellular	U35462
*S. lycopersici*	*B2Tom*	Tomatinase	Extracellular	AAB08445
*Stagonospora avenae*	*savBGL1*	Beta-glucosidase	Extracellular	AAT95376
*B. cinerea*	*sap1*	Avenacinase	Extracellular	XM_024695232
*N. vasinfecta*	*sdn1*	Saponin hydrolase	Extracellular	42491247
*F. oxysporum*	*tom1*	Tomatinase	Extracellular	AJ012668
**Isothiocyanates**				
*S. sclerotiorum*	*SsSaxA*	ITC hydrolases	Peroxisome	APA09264
*A. brassicicola*	*AbGST1*	Glutathione S-Transferase	Cytoplasmic	AY987487

Putative localizations predicted using SignalP and DeepLoc softwares.

*Genetic information for this protein is unavailable, but protein characterization points toward secretion into extracellular space (Turbek et al., 1992).

### Isoflavones

Isoflavones are a class of phenylpropanoids primarily known for their importance in legume defense to pathogens (Ahuja et al., 2012). Kievitone is a member of this class which accumulates, along with a range of other isoflavonoid phytoalexins, during fungal infection of *P. vulgaris* (Van Etten and Smith, 1975; Smith et al., 1984). Early studies determined that *F. solani* detoxified kievitone through a kievitone hydratase (KHase) reaction and a clear association between KHase activity and virulence on common bean has been readily observed (Kuhn and Smith, 1979; Smith et al., 1982). *F. solani* isolates/mutants lacking this activity were non-pathogenic, highlighting its critical importance during infection of *P. vulgaris* (Smith et al., 1984, 1982). The gene encoding kievitone hydratase was first cloned from *F. solani* f. sp. *phaseoli* and its function in converting kievitone to kievitone hydrate was confirmed through heterologous expression (Daoxin et al., 1995). Putative homologs of *khs* have been identified in other non-pathogenic *Fusarium* spp., suggesting that this gene is not a solely capable of granting virulence to *P. vulgaris* (Daoxin et al., 1995). More recently the gene encoding KHase in *N. haematococca* (*NhKHS*) was cloned and characterized through recombinant expression (Engleder et al., 2018). Purified NhKHS appears capable of acting on kievitone and several other flavonoid compounds, suggesting that this enzyme may be capable of detoxifying a wider range of PDCs than originally thought (Engleder et al., 2018). To date, *khs* deletion mutants have yet to be generated in any species, thus the involvement of other genes in this process cannot be excluded.

While no other isoflavone detoxification mechanisms have been specifically characterized, metabolic profiling has shown that other isoflavones, including the major isoflavones genistein and daidzein, can be metabolized by a number of other fungal pathogens (Table 1).

### Other Flavonoids and Stilbenoids

Flavonol-type PDCs, such as quercetin and kaempferol, are found in a wide range of plants and demonstrate antimicrobial activity both in their free and glycosylated forms (Lygin et al., 2009; Sanzani et al., 2009; Chen et al., 2019). Quercetinase activity has been observed in a number of pathogens and quercetin dioxygenases (QDOs) responsible for this activity have been characterized in *Penicillium olsonii*, *Sclerotinia sclerotiorum*, and *Verticillium dahlia* (Tables 1, 2). While the enzyme from *V. dahlia*, VDQase, is putatively cytoplasmic, those found in the other two pathogens appear to be secreted, possibly as a dual mechanism that simultaneously detoxifies the compound and subvert the capacity of the host to produce glycosylated flavonols, such as rutin (Table 2). The detoxification of rutin requires the cleavage of the compounds sugar moiety through a proposed fungal rutinase, which subsequently releases the mycotoxic quercetin, thus implicating quercetinases in tolerance to both flavonols and their cognate flavonoid glycosides (El Hadrami et al., 2015). Interestingly, QDO activity has been primarily observed in broad-host range pathogens, whereas detoxification of other flavonoids, such as sakuranetin from rice, appears to utilize a distinct detoxification pathway in the more specialized pathogen *Magnaporthe oryzae* (Table 1).

Stilbenoids, another class of inducible PDC, help to modulate plant immunity to phytopathogens as well as UV and ozone stress (Chong et al., 2009). The most studied stilbenoid is the grapevine phytoalexin resveratrol, which has an important role in grapevine resistance to the bunch rot pathogen *Botrytis cinerea*. While resveratrol is suppressive to *B. cinerea* growth, it is well established that the pathogen is capable of oxidizing the phytoalexin *in vitro* and this activity has been attributed to a secreted laccase, Bclcc2 (Schouten et al., 2002). Counterintuitively, this laccase acts as a profungicide metabolizing resveratrol to a more toxic metabolite, and gene knockout mutants of *Bclcc2* are both resistant to resveratrol and maintain full virulence on grapevine, leaving the role of this enzyme in pathogenesis unclear (Schouten et al., 2002). A more linear story of stilbenoid detoxification can be seen in the spruce pathogen *Endoconidiophora polonica.* After being deposited into a spruce tree by its bark-beetle vector, the host responds to invasion by producing antifungal compounds such as the stilbenoid astringin and the flavan-3-ol catechin. *E. polonica* detoxifies these compounds using a group of four catechol dioxygenases (CDOs), one of which is secreted and operates at the forefront of infection, and the other three of which operate intracellularly (Wadke et al., 2016) (Table 2).

### Indoles

Indoles are a class of PDC known for their role in defense against biotic stress in cruciferous (brassica) vegetables (Pedras and Ahiahonu, 2005). Detoxification of indoles has been observed in broad-host range necrotrophs including *Sclerotinia sclerotiorum* and *B. cinerea* as well as the more specialized Brassicaceae pathogen *Leptosphaeria maculans*, albeit through seemingly distinct mechanisms (Table 1). A wide range of anti-fungal indoles have been characterized, yet only proteins with the capacity to degrade a single one, the phytoalexin brassinin, have been properly characterized (Table 1). Brassinin hydrolases (BHs), seen in the crucifer pathogens *Alternaria brassicicola* and *L. maculans*, transform brassinin to the less toxic indolyl-3-methanamine (Pedras et al., 2009). In addition to its BH activity, *L. maculans* also detoxifies brassinin through an inducible brassinin oxidase (BO) (Table 1) (Pedras et al., 2008). Both enzymes demonstrate a surprisingly narrow substrate range and are not only incapable of transforming related indoles, such as cyclobrassinin, but in many cases are competitively inhibited by them (Pedras et al., 2008, 2009). This inhibition is intriguing as brassinin is a metabolite required for the production of other antifungal indoles, meaning BH/BO activity may be achieving multiple goals simultaneously: (1) degrading antifungal brassinin in the host, (2) preventing the production of other antifungal indoles, and (3) preventing the production of BH/BO inhibitors.

Rather than oxidation/hydrolysis, *S. sclerotiorum* appears to detoxify brassinin using a rather uncommon glycosylation reaction in which brassinin is transformed through the conjugation of a glucose molecule. The enzyme responsible for this transformation, SsBGT1, appears to have a similar substrate specificity and induction pattern to other brassinin detoxifying enzymes, but inhibition by natural indoles has not been reported (Table 2) (Sexton et al., 2009). Metabolism of other indoles by fungal pathogens is summarized in Table 1.

### Terpenes/Terpenoids

Unlike many of the above-described classes of PDC, which are formed as downstream products of the shikimate pathway, terpenoids are generated through either the mevalonate (MVA) or methylerythritol 4- phosphate (MEP) pathways in plants. Despite the broad range of terpenoids known to operate in chemical defense and the number of phytopathogens known to metabolize terpenoids *in vitro*, the only fungal enzymes characterized in terpenoid detoxification are from the pine pathogen *Grosmannia clavigera* (Table 1) (Wang et al., 2014). Multiple enzymes were involved in the metabolic utilization of monoterpenes, primarily the antifungal limonene, but only two Baeyer–Villiger monooxygenases were implicated specifically in enzymatic detoxification (Table 2) (Wang et al., 2014).

## Enzymatic Detoxification of Non-Inducible PDCs

Non-inducible PDCs are defined in this review as low molecular weight antimicrobial compounds that are pre-formed in healthy plant tissue rather than being produced in response to pathogen invasion. This category includes many phytoanticipins, but excludes those which are both constitutively present in healthy tissue and are also induced in response to infection, examples of which include resveratrol and maackiain (Van Etten et al., 1994; Wang et al., 2013). Non-inducible PDCs are stored in either an active form or as inactive precursors which are activated in response to tissue damage (Tiku, 2020). As this damage is an inevitable result of fungal necrotrophy, these compounds provide protection that is less easily subverted by fungal effectors targeting plant defense pathways than phytoalexins might be. The following section will focus on the detoxification of important classes of non-inducible PDCs by fungal pathogens. A more complete summary of fungal metabolism of these compounds can be found in Table 3.

**TABLE 3:** Metabolism of non-inducible PDCs by fungal necrotrophs and hemibiotrophs.

Class	Compound	Fungal species	Conversion product	GEC	Citation
**Cyanide**	Cyanide	*L. maculans*	Formamide	+	Sexton and Howlett, 2000
		*Gloeocercospora sorghi*	Formamide	+	Wang and Van Etten, 1992; Wang et al., 1999
		*Stemphylium loti*	Formamide	−	Nazlya et al., 1983
		*F. moniliforme*	Formamide	−	Nazlya et al., 1983
		*C.graminicoal*	Formamide	−	Fry and Evans, 1977
		*Helminthosporium maydis* race T	Formamide	−	Fry and Evans, 1977
		*H. turcicum*	Formamide	−	Fry and Evans, 1977
		*Macrophomina phaseoli*	Formamide	−	Fry and Evans, 1977
		*Mycoleptodiscus terrestris*	Formamide	−	Fry and Evans, 1977
		*Phoma* spp.	Formamide	−	Fry and Evans, 1977

**Saponins**	Avenacoside A	*Stagonospora* avenae	Deglycosylated derivative	+	Morrissey et al., 2000
		*Septoria avenae*	Deglycosylated derivative	−	Wubben et al., 1996
	Avenacoside B	*Stagonospora avenae*	Deglycosylated derivative	+	Morrissey et al., 2000
		*Septoria avenae*	Deglycosylated derivative	−	Wubben et al., 1996
	Avenacin A-1	*Gaeumannomyces graminis vat. tritici*	Deglycosylated derivative	+	Bowyer et al., 1995
	a-tomatine	*B. cinerea*	β-1-Tomatine	+	Quidde and Peter, 1999
		*F. oxysporum* f. sp. *lycopersici*	Tomatidine	+	Roldán-arjona et al., 1999
		*F. solani*	Tomatidine	−	Lairini and Ruiz-rubio, 1998
		*S. lycopersici*	β-2-Tomatine	+	Martin-Hernandez et al., 2000; Sandrock and Van Etten, 2001
		*A. alternata*	Unknown product	−	Oka et al., 2006
		*Corynespora cassiicola*	Unknown product	−	Oka et al., 2006
		*C.coccodes*	Unknown product	+	Sandrock and Van Etten, 2001
	α-Solanine	*G. pulicaris*	γ-Solanine	−	Weltring et al., 1997
	α-Chaconine	*G. pilicaris*	β-2-Chaconine	−	Weltring et al., 1997
	Soyasaponin I	*Neocosmospora vasinfecta* var. *vasinfecta*	Soyasapogenol B Triose	+	Watanabe et al., 2004
	Soyasaponin II	*N.vasinfecta* var. *vasinfecta*	Deglycosylated derivative	+	Watanabe et al., 2004

**Benzoxazinoids**	2-Benzoxazolinone (BOA)	*F.verticilloides*	N-(2-hydroxyphenyl) malonamic acid (HPMA)	+	Glenn et al., 2002
		*F. graminearum*	N-(2-hydroxyphenyl) malonamic acid (HPMA)	+	Baldwin et al., 2019
		*F. pseudograminearum*	N-(2-hydroxyphenyl) malonamic acid (HPMA)	+	Kettle et al., 2015
		*F. culmorum*	N-(2-hydroxyphenyl) malonamic acid (HPMA)	−	Friebe et al., 1998
		*G. graminis*	N-(2-hydroxyphenyl) malonamic acid (HPMA)	−	Friebe et al., 1998
	6-Methoxy-2-benzoxazolinone (MBOA)	*F.verticilloides*	N-(2-hydroxy-4-methoxyphenyl (HMPMA)	+	Glenn et al., 2002
		*F. pseudograminearum*	N-(2-hydroxy-4-methoxyphenyl (HMPMA)	+	Kettle et al., 2015
		*G. graminis*	N-(2-hydroxyphenyl) malonamic acid (HPMA)	−	Friebe et al., 1998

**ITCs**	Benzyl isothiocyanates	*A. brassicicola*	S-glutathionylated product	+	Sellam et al., 2006
	Allylisothiocyanates	*A. brassicicola*	S-glutathionylated product	+	Sellam et al., 2006
	4-methylsulfinylbutyl ITC	*S. sclerotiorum*	4-Methylsulfinylbutyl acetamide	+	Chen et al., 2020
			4-MethylsulfinylbutylN-acetylcysteine	−	Chen et al., 2020

*GEC,gene or enzyme characterized?*

### Hydrogen Cyanide

Cyanogenic plants are a broad group, including thousands of individual plant species and >120 plant families, that are characterized by the presence of cyanogenic glycosides in their tissue (Tiku, 2020). Tissue damage leads to the release of plant glycoside hydrolases which cleave the sugar moiety, converting these glycosides to cyanohydrins, which are subsequently transformed to hydrogen cyanide (HCN) by hydroxynitrile lyases (Tiku, 2020). Pathogens of cyanogenic plants are all capable of tolerating HCN and a single mechanism, cyanide hydration, appears to be an incredibly conserved detoxification mechanism used across fungal pathogens of these plants (Table 3) (Fry and Evans, 1977). Cyanide hydratases (CHT) responsible for this activity have been characterized in two pathogens, *L. maculans* and *Gloeocercospora sorghi*, both of which are putatively cytoplasmic proteins (Table 2). These CHTs efficiently convert HCN to formamide, and CHT knockouts in *G. sorghi* were far more sensitive to potassium cyanide, an inducer of CHT activity (Wang et al., 1999).

### Saponins

Saponins are compounds containing a triterpenoid steroid, or steroidal glycoalkaloid bound to one or more sugar chains and have been utilized for hundreds of years as a surfactant, but have gained notoriety in recent decades for their antimicrobial properties (Osbourn, 1996). Prior to pathogen attack saponins are kept in an inactive form with multiple sugar chains bound to the alkaloid and are spatially separated from the hydrolytic enzymes needed to activate them. In response to tissue damage, this compartmentalization which maintains saponin precursors in vacuoles and hydrolytic enzymes in plastids breaks down, allowing the enzymes to cleave one or more sugars to generate bioactive saponins. Unlike most other phytoanticipins that are fully deglycosylated during activation, the antifungal activity of saponins are dependent on their sugar moieties (Osbourn, 1996).

Nearly all pathogens of saponin-containing plants depend on some detoxifying activity to tolerate these compounds during infection, and all characterized saponin detoxification enzymes operate through the cleavage of one or more sugar molecules from the compounds sugar chain (Osbourn, 1996) (Tables 2, 3). The first discovered of these enzymes, an avenacinase termed Avn, is used by the Take-All fungus *Gaeumannomyces graminis* to detoxify oat saponins and is seemingly a singular determinant in the pathogens capacity to infect oats (discussed further in section “The Role of PDC Tolerance in Fungal Host Range”) (Bowyer et al., 1995). Additional oat pathogens, *Stagonospora avenae* and *Septoria avenae*, encode multiple saponin hydrolases (SHs) which cleave distinct bonds in the sugar chains of these oat saponins, referred to as avenacosides, and are primarily present in oat-infecting isolates, but absent in those isolated from saponin-deficient wheat (Wubben et al., 1996; Morrissey et al., 2000) (Table 3).

While SHs were originally thought to be somewhat specific to oat pathogens, studies into the saponin detoxifying activity of the tomato pathogen *Septoria lycopersici* found that it utilized a secreted tomatinase enzyme (Tom) with homology to Avn from *G. graminis* (Osbourn et al., 1995). Tomatinases, named for their ability to degrade the important tomato saponin α-tomatine, are found in a wide range of tomato-infecting fungal pathogens (Tables 2, 3). The presence of avenacinases, tomatinases, and other SHs is highly correlated with the ability to infect saponin-containing plants, but as many pathogens contain multiple distinct SHs that are active during pathogenesis, the importance of individual enzymes in virulence is difficult to discern through simple gene disruption experiments (Quidde and Peter, 1999; Dufresne et al., 2000). Ectopic expression of the tomatinase-deficient pathogen *N. haematococca* MPVI with a β2-tomatinase gene from *S. lycopersici* allowed it to infect green tomato fruit, confirming that saponin detoxifying enzymes can act as singular determinant of pathogenicity on certain hosts (Sandrock and Van Etten, 2001). Although SHs appear to be primarily responsible for tolerance to plant saponins, evidence has also been presented to suggest that saponin degradation products may modulate plant immunity as well (discussed in section “Detoxification and Plant Defense Signaling”).

### Benzoxazinoids

Benzoxazinoid-type phytoanticipins are indole-derived compounds common across cereal crops and are broadly toxic to pathogens and pests (Tiku, 2020). Similar to other phytoanticipins, benzoxazinoids are stores in a glycosylated form in plant vacuoles and are converted to their active form by plant glucosidases in response to tissue damage (Glenn and Bacon, 2009). Detoxification of these compounds has been primarily studied in cereal-infecting *Fusarium* spp. and relies on a two-step process to fully metabolize benzoxazolin-2(3H)-one (BOA) and 6-methoxybenzoxazolin-2(3H)-one (MBOA), the major antifungal benzoxazinoids found in maize and wheat (Table 3). The genes involved in this transformation are a metallo-β-lactamase referred to as *MBL1*, or *Fdb1*, and an arylamine *N*-acetyltransferase referred to at *Nat1*, or *Fdb2* (Table 2). MBL1 catalyzes an initial conversion of BOA to 2-amino-phenoxazin-3-one (2-APO), which is subsequently converted to the negligibly mycotoxic *N*-(2-hydroxyphenyl) malonamic acid (HPMA) by Nat1. The specific requirement of benzoxazinoid detoxification in virulence is unclear, as genetic knockouts of these genes in multiple *Fusarium* spp. have resulted in conflicting results, with clear virulence defects observed in *Fusarium pseudograminearum*, but none in *Fusarium graminearum* or *Fusarium verticillioides* (Glenn et al., 2002; Kettle et al., 2015; Baldwin et al., 2019).

### Isothiocyanates

Glucosinolates are amino acid derived plant compounds that are converted into toxic isothiocyanates (ITCs) in response to tissue damage. These compounds are found primarily in cruciferous plants, and ITCs are potent in suppressing both invasion by fungal/bacterial pathogens and herbivory by pests (Bednarek et al., 2009). The detoxification of ITCs has been well characterized in animals and is typically catalyzed by glutathione *S*-transferases (GSTs), a class of enzyme that can conjugate glutathione (GSH) to various xenobiotic substrates. The crucifer pathogen *A. brassicicola* detoxifies these compounds using a similar mechanism by producing a specific GST, AbGST1, in response to ITC exposure (Sellam et al., 2006). Other GSTs in *A. brassicicola* have also been characterized and appear to be important in pathogenesis, but their specific role in ITC detoxification is unclear (Calmes et al., 2015).

Evidence suggests that the broad-host range pathogen *S. sclerotiorum* also uses GST activity in the detoxification of ITCs to a minor extent, but the major detoxification mechanism (100-fold greater) appears to be through hydrolytic degradation. An ITC hydrolase, SsSaxA, is credited with this activity and is capable of efficiently degrading the aliphatic and aromatic ITCs of the host *Arabidopsis thaliana* (Chen et al., 2020).

### Phenylpropanoid Derivatives

Derivatives of the phenylpropanoid pathway, including ferulic and cinnamic acid, are aromatic compounds necessary for the generation of structural lignin as well as other physiological processes (Mäkelä et al., 2015). While not typically classified as phytoanticipins due to their pivotal role in plant physiology, they share many characteristics including a constitutive presence in plant tissue and antifungal activity (Pacheco et al., 2018). It has been demonstrated that the accumulation of these compounds in plant tissue is important for soybean resistance to the broad host-range necrotroph *S. sclerotiorum* and the capacity to metabolize these compounds to some extent is a rather ubiquitous feature of filamentous fungi (Mäkelä et al., 2015; Ranjan et al., 2019). To our knowledge the mechanism for this metabolization of phenylpropanoid derivatives has not been characterized in any fungal species despite the necessity of such a process in colonizing plant tissue.

## Toxin Efflux

While degradative detoxification of PDCs has received a large amount of attention in recent years, it has long been known that some non-degradative mechanism of fungal tolerance to PDCs exists as well (Denny and Van Etten, 1983a, b). Despite that, it was not until the discovery of the first multidrug resistance (MDR) transporters in *Saccharomyces cerevisiae*, SNQ2 and PDR5 (Servos et al., 1993; Balzi et al., 1994), and the subsequent description of the first ATP-binding cassette (ABC) transporter in the rice pathogen *M. oryzae* that a true mechanism for this activity was described (Urban et al., 1999).

Contrary to the enzymatic detoxification of PDCs, toxin efflux operates through the activity of membrane bound substrate transport proteins that work to either export or sequester toxic compounds. Broadly, these proteins can be separated into ABC transporters that operate using energy from ATP hydrolysis and major facilitator superfamily (MFS) transporters which utilize a chemiosmotic gradient generated across the membrane to move substrate (Pao et al., 1998). As plant hosts typically possess a repertoire of chemically related PDCs, efflux proteins often have a broad substrate binding affinity and are thus referred to as pleiotropic and/or multidrug resistance proteins (PDR and/or MDR). These proteins are most often transcriptionally regulated and thus some insight into their substrate range can be gained through the accumulation of their RNA transcripts upon exposure to various compounds (Schoonbeek et al., 2001; Semighini et al., 2002; Lee et al., 2005; Gupta and Chattoo, 2008).

True to their name as pleiotropic drug resistance proteins, individual efflux transporters are often shown to be active against a range of chemically distinct compounds (Roohparvar et al., 2007). This broad range of activity has been functionally validated in multiple transporters within the wheat pathogen *Zymoseptoria tritici*, where ectopic expression of these transporters in yeast conferred increased tolerance to a variety of antifungal compounds. Surprisingly, though, genetic knockouts of these transporters often show no reduction in virulence (Table 4). Multiple theories have been discussed to address this, the most common being a functional redundancy between transporters with similar substrate ranges. Substrate redundancy across a range of toxic compounds has been observed in the broad host-range pathogen *B. cinerea*, the wheat pathogen *Z. tritici*, and the saprotroph *Aspergillus nidulans* (Semighini et al., 2002; Schoonbeek et al., 2003; Zwiers et al., 2003). This redundancy has been characterized in both plant and animal pathogens and often requires multiple transporters to be knocked out in a single mutant to see the expected increase in sensitivity to a suspected substrate (Sanglard et al., 1997; Hayashi et al., 2002).

**TABLE 4:** Putative efflux transporters involved in fungal tolerance to plant defense compounds.

Species	Gene name	Transporter type	Putative substrate/ligand	PCV?	Accession
*Alternaria alternata*	*AaMFS54*	MFS	Diverse range of xenobiotics	Yes	CP061877
*Botrytis Cinerea*	*BcAtrA*	ABC	Cycloheximide, catechol, eugenol	No	XM_001558433
	*BcAtrB*	ABC	Diverse range of xenobiotics	Yes	XM_024696626
	*BcAtrD*	ABC	DMI fungicides, cycloheximide, eugenol	No	XM_001555199
	*BcAtrF*	ABC	Resveratrol	NT	AAF64440
	*BcAtrG*	ABC	Cycloheximide, tomatin, multiple phenolics	NT	CAB92309
	*Bmr1*	ABC	Multiple fungicide classes, resveratrol	NT	XM_001561290
	*Bmr3*	ABC	Multiple fungicide classes, resveratrol	NT	XM_024693911
	*mfsG*	MFS	Isothiocyanates	Yes	XM_024693262
	*Bcmfs1*	MFS	Camptothecin, DMI fungicides	No	AF238225
	*Bcmfs2*	MFS	Cycloheximide, tomatin	NT	XP_024546409
	*Bcmfs4*	MFS	Cycloheximide, psoralen, multiple phenolics	NT	XM_024691536
*Clarireedia Jacksonii*	*ShPDR1*	ABC	Multiple fungicide classes	NT	KJ128076
*Colletotrichum acutatum*	*CaABC1*	ABC	Multiple fungicide classes, Hygromycin	NT	KM264299
*Gibberella pulicaris*	*Gpabc1*	ABC	Rishitin	Yes	AJ306607
*Grosmannia clavigera*	*GcABC-G1*	ABC	Monoterpenes	Yes	EFX05787
*Magnaporthe grisea*	*ABC1*	ABC	Unknown	Yes	XM_003717474
	*ABC2*	ABC	DMI fungicides, Camptothecin, cycloheximide	No	AB091269
	*ABC4*	ABC	Resveratrol, miconazole, cycloheximide	Yes	XM_003717966
*Zymoseptoria tritici*	*MgAtr1*	ABC	Diverse range of xenobiotics	No	XM_003857588
	*MgAtr2*	ABC	Diverse range of xenobiotics	No	XM_003848105
	*MgAtr4*	ABC	Diverse range of xenobiotics	Yes	XM_003848300
	*MgAtr5*	ABC	Berberine, camptothecin	No	XM_003852443
	*MgMFS1*	MFS	Azoles, plant alkaloids, mycotoxins	No	XM_003850512
*Nectria haematococca*	*NhABC1*	ABC	Pisatin, Rishitin	Yes	HM106507
	*PEP5*	MFS	Unknown	Yes	XM_003044178
*Penicillium digitatum*	*PMR1*	ABC	DMI fungicides, phloretin, camptothecin, oligomycin	No	AB010442
	*PMR5*	ABC	Diverse range of xenobiotics	No	AB060639
	*PdMfs1*	MFS	DMI fungicides, Plant metabolites suspected	Yes	AM412556
	*PdMFS1*	MFS	Prochloraz	Yes	GU124565
	*PdMFS2*	MFS	Prochloraz	Yes	GU228489

*PCV, positively correlated with virulence.*

The importance of a distinct transporter in infection is dependent on the hosts antimicrobial repertoire, so an additional explanation is that it may be necessary to screen multiple hosts before finding one in which a given transporter is pivotal to pathogenicity. In the case of the *B. cinerea* ABC transporter *BcAtrB*, knockout mutants showed no reduction in virulence on basil, but were compromised during infection of *A. thaliana* and grapevine (Schoonbeek et al., 2001, 2003; Stefanato et al., 2009).

Although there are certainly incidences in which the role of a putative efflux transporter is vague, many of those characterized in necrotrophic and hemibiotrophic pathogens have a clear role in virulence (Table 4). This role is often elucidated through a mixture of *in vitro* sensitivity and disease assays to establish a likely PDC substrate and quantify the importance during host colonization, but it’s typically difficult to prove causation between the loss of specific efflux activity and a reduction in virulence. This concern has been raised given that some transporters which contribute to MDR may do so as a side-effect of primary roles in lipid transport, plasma membrane integrity, and/or fungal development (Stergiopoulos et al., 2003; Gupta and Chattoo, 2008; Shahi and Moye-Rowley, 2009). An optimal method to establish this causation is by showing that efflux transporter mutants with a virulence defect on wild-type host plants are fully capable of infecting hosts deficient in the PDC substrate of interest. This has only been demonstrated in *BcAtrB* and *mfsG* from *B. cinerea*, which export the phytoalexin camalexin and ITC-type phytoanticipins, respectively. In both of these cases camalexin and ITC-deficient plants were generated in *A. thaliana*, an organism that benefits from genetic tractability, and thus, similar evidence remains elusive in other pathosystems (Stefanato et al., 2009; Vela-corcía et al., 2019).

While the importance of efflux in fungal tolerance to a number of PDCs is apparent, an inherent limitation to this mechanism is that it can only act on PDCs that target components within the fungal cell. Accordingly, there are a number of transporters with apparent activity against resveratrol, a plant stilbenoid thought to target cellular respiration, but none that act against plant saponins, which target the plasma membrane (Osbourn, 1996; Schoonbeek et al., 2001, 2003; Caruso et al., 2011; Adrian and Jeandet, 2012). Thus, in order to cope with a broader range of PDCs, efflux transporters often work in tandem with other detoxification mechanisms.

## Working in Concert: Multiple Mechanisms of Toxin Tolerance

Cooperative relationships between efflux transporters and other detoxification mechanisms is expected, and have been described in multiple pathosystems, including *G. clavigera* (Wang et al., 2012, 2014), *B. cinerea* (Schouten et al., 2008), and *N. haematococca* (Han et al., 2001; Coleman et al., 2011b). In these relationships the efflux activities of the transporters activate very early during host colonization to avoid the accumulation of host PDCs in fungal hyphae, this is coupled with a subsequent activation of enzymes to detoxify compounds through chemical modification. Such a system has been observed on a broader scale by the fungal pathogen *S. sclerotiorum* during infection of soybean. A time-course transcriptomic analysis of this infection showed an enrichment of ABC and MFS transporters putatively involved in drug transport during early infection and a subsequent increase in genes putatively involved in degradative detoxification of xenobiotics during later timepoints (Westrick et al., 2019).

In addition to the broad use of detoxification enzymes, some fungi may also modify the infection court in a coordinated approach. *B. cinerea* has a repertoire of enzymes that are known to be involved in the degradation of saponins (Quidde and Peter, 1999). Interestingly, *B. cinerea* also acidifies host tissue during infection to facilitate disease, this increasingly acidic environment causes saponins to lose their antifungal activity (Kunz et al., 2006). Thus, the acidification of host tissue in *B. cinerea* may decrease the efficacy of plant saponins and allow for other degradative mechanisms to finish the job, through a similar coordinated relationship as that described above. This ability to reduce saponin efficacy through acidification may explain why the closely related *S. sclerotiorum*, a particularly prolific producer of oxalic acid, has no clear homolog of genes coding for some common saponin detoxifying enzymes tomatinase or avenacinase in its genome (Figure 1). Thus, *S. sclerotiorum*’s incredibly broad range may be owed to its ability to create an environment that renders plant chemical defenses powerless.

Another tool at the disposal of fungal pathogens noted in this review, is to simply deploy multiple enzymes that act in concert to deal with specific plant metabolites. It has been noted on multiple occasions that genes putatively involved in fungal detoxification are often conserved in gene clusters, similar to the secondary metabolite gene clusters found in most fungal genomes (Gluck-thaler and Slot, 2018). This has led to the assertion that these genes may be operating in tandem to efficiently metabolize host PDCs, but to date, only individual genes in these clusters have been credited with a role in detoxification, leaving the purpose of this gene clustering elusive (Glenn et al., 2016; Gluck-thaler and Slot, 2018).

## Metabolism or Detoxification: Maybe Both?

Mechanisms for detoxifying PDC’s in phytopathogenic fungi are, at their core, responsible for overcoming plant immunity and facilitating the metabolism of host tissue. Given this fact it is tempting to draw a line between detoxification and metabolism, when in fact data suggests that they are far more interconnected than originally thought. An interesting example of this can be seen in the D-galacturonic acid (GalA) metabolic pathway, which is highly conserved in among pectin-degrading filamentous fungi (a category encompassing nearly all fungal necrotrophs and hemibiotrophs) (Martens-uzunova and Schaap, 2008). GalA is the primary constituent of pectin in plants and is degraded by fungal pathogens through a four step metabolic pathway into glycerol and pyruvate (Martens-uzunova and Schaap, 2008). While GalA is often considered a simple nutrient source for fungi, it is also capable of actively inhibiting the growth of the non-pathogenic yeast *S. cerevisiae*, indicating that the compound may have some antimicrobial properties (Huisjes et al., 2012). The components of the GalA metabolic pathway have been studied in multiple necrotrophic pathogens and individual gene knockouts of each component of this pathway have been shown to display reduced virulence on hosts rich in GalA (Martens-uzunova and Schaap, 2008; Zhang et al., 2011; Wei et al., 2020). Rather than a metabolic defect, this loss in virulence is due to the accumulation of suppressive amounts of GalA and its derivatives, compounds which could act as either carbon sources or toxins depending on the pathogen’s capacity to metabolize them (Zhang and van Kan, 2013).

Similar overlaps between metabolism and detoxification have been seen in the spruce pathogen *E. polonica* and the pine pathogen *G. clavigera*. Both pathogens must detoxify antimicrobials, primarily stilbenoids and terpenoids, induced by their beetle vectors at the site of infection (Franceschi et al., 2005; Keeling and Bohlmann, 2006). Rather than simply degrading these compounds, both *E. polonica* and *G. clavigera* have evolved multiple enzymes that facilitate the degradation and metabolic utilization of their respective PDCs (Wang et al., 2014; Wadke et al., 2016). *G. clavigera* is among the most aggressive fungal pathogens to pine trees in North America due its perceived tolerance and utilization of host monoterpenes to a greater degree than other pine ophiostomatoid fungi (Alamouti et al., 2011; Wang et al., 2014). The pathogen *Armillaria mellea* is capable of metabolizing the antifungal PDC genistein into multiple less toxic derivatives which can be utilized as carbon sources (Curir et al., 2006). Additionally, *L. maculans* can use formamide, the result of cyanide hydratase detoxification of HCN, as a sole carbon source (Sexton and Howlett, 2000).

The function of saponin hydrolase enzymes is another potential example of this crossover, they are used by many phytopathogenic fungi to detoxify plant saponins, a class of molecules typically consisting of a hydrophobic aglycone with appended sugar molecules. These enzymes reduce the antifungal activity of saponins through the removal of sugar molecules from their glycosyl chains, releasing glucose and/or other sugars in the process (Osbourn, 1996). While the metabolic utilization of these released sugars has not been specifically addressed to our knowledge, it would be expected if these sugars were used by the pathogen as a carbon source. Many of these enzymes belong to the glycoside hydrolase family 3 (GH3) family of proteins and are related to cellobiose degrading enzymes used to release sugars from plant cell walls during fungal necrotrophy (Osbourn et al., 1995).

## The Role of PDC Tolerance in Fungal Host Range

Tolerance to specific plant defense compounds, be it through detoxification, efflux, or target modification, is an absolute necessity in a pathogen’s capacity to infect a host and is therefore a major determinant in its host range. The simplest example of this phenomenon is seen in the take-all pathogen *G. graminis*, which can infect a range of cereal crops. Varieties of *G. graminis* differ in their ability to infect oat and these differences appear to be largely defined by their capacity to detoxify saponins, which are found in most oats but are absent in many other cereal species (Bowyer et al., 1995). This detoxification activity was attributed to a specific enzyme (Avn) in the oat-infecting variety, *G. graminis* var. *avenae*, that effectively detoxifies the oat saponin Avenacin A-1. Avn acts as a singular determinant of pathogenicity on oats, with Avn-minus mutants losing the ability to infect oats but retaining virulence on saponin-deficient species including wheat (Bowyer et al., 1995). The potential for a similar host-range expansion can be seen from the *S. lycopersici* tomatinase, B2Tom, which efficiently hydrolyzes saponins found in green tomatoes (Table 2). When *N. haematococca* was transformed to express this protein, it gained the ability to infect green tomatoes, a feature that WT-strains lack (Sandrock and Van Etten, 2001). Therefore, the host range of some closely related fungi, or even strains of the same species, can differ due to minor changes to their detoxification repertoires.

Pisatin detoxification also appears to be important in the ability for Fusarium species to infect pea, as pisatin is one of its primary defense compounds induced during fungal infection and tolerance to pisatin appears critical for host tissue colonization (Cruickshank and Perrin, 1962; Delserone et al., 1999). The primary enzyme for pisatin degradation in *F. solani* is a pisatin demethylase (PDA), but unlike Avn in *G. graminis*, PDA-minus mutants do not fully lose pathogenicity, likely due to multiple mechanisms of tolerance to pisatin (Table 2) (Coleman et al., 2011b). Despite this, non-pathogenic strains of *F. solani* and the maize pathogen *Cochliobolus heterostrophus* which are not pea pathogens, gained pathogenicity through ectopic expression of PDA. This suggests that pisatin may be a critical driver of non-host resistance in pea and PDA expression is alarmingly enough to overcome this barrier (Schafer et al., 1989; Ciuffetti and Van Etten, 1996).

In the case of broad-host range pathogens detoxification is critically important as the fungus must maintain a repertoire of detoxification genes to tolerate the wide array of defense compounds being brought to bear in different hosts. These genes hold differential importance on distinct hosts as can be seen in the broad-host range pathogens *S. sclerotiorum* and *B. cinerea* (Figure 1). Both pathogens contain genes encoding putative cyanide hydratases for the detoxification of HCN and induction of this gene is likely regulated by the HCN content of the host, as cyanide hydratase expression has a dose-dependent relationship to cyanide content in other fungal systems (Barclay et al., 2002). Such a pattern is seen in the ITC detoxifying gene SsSaxA, which is only induced in the ITC containing crucifers *A. thaliana* and *Brassica napus*. The major toxin efflux transporters in *B. cinerea* are seemingly utilized to a much greater degree during infection of tomato than grapevine and inversely the laccase *Bclcc2* which metabolizes the grapevine phytoalexin resveratrol is only induced during infection of grapevine (Figure 1). This transcriptional plasticity is a hallmark of broad-host range pathogens and the large number of detoxification mechanisms retained in their genome is likely a necessity for their lifestyle. This is similarly seen when comparing isolates of *Alternaria alternata*, a broad-host range necrotroph that retains tomatinase activity across a wide range of isolates regardless of their primary host, and *Corynespora cassiicola*, a comparatively narrow host range pathogen which has only retained tomatinase activity in specific tomato-infecting strains (Oka et al., 2006). Though it is convenient to utilize model plants to analyze virulence factors and pathogenic attributes of broad-host necrotrophs, considering the genomic plasticity seen in some of these pathogens, it is prudent to study their pathogenic development across multiple hosts.

An expansion of host range has been demonstrated through the horizontal gene transfer (HGT) of host-specific toxins in wheat pathogens, and a similar transfer of detoxification genes may have also allowed for the expansion of other pathogens to novel hosts (Mcdonald et al., 2019). Evidence suggests that phytopathogenic oomycetes gained cyanide hydratase genes through HGT from true fungi and benzoxazinoid detoxifying gene clusters appear to have spread through a similar mechanism from *Fusarium* spp. to species of *Colletotrichum* and *Aspergillus* (Richards et al., 2011; Glenn et al., 2016).

## Detoxification and Plant Defense Signaling

In most plant–pathogen interactions the primary purpose of PDC detoxification appears to be the protection of the pathogen, but in some cases the metabolism of PDCs may play a larger role in subverting host defenses. This is highlighted by the tomatinase enzyme from *S. lycopersici* (Tom), which is known to hydrolyze the tomato phytoanticipin α-tomatine (Table 2) (Martin-Hernandez et al., 2000). An examination of tomatinase activity in *Nicotiana benthamiana* found evidence that the enzyme appears to suppress the host’s broader immune response in addition to its role in saponin detoxification (Bouarab et al., 2002). Indeed β2-tomatine, the hydrolysis product of α-tomatine, is capable of directly suppressing the induction of multiple plant defense genes (PR1, PR5, and AOS) during infection (Bouarab et al., 2002). β2-Tomatine also facilitated the growth of the bacterial pathogen *Pseudomonas syringae* pv. *tabaci in planta* (Bouarab et al., 2002). Although less well characterized, the *Verticillium dahliae* quercetinase enzyme, VDQase, is also suggested to manipulate the host phytohormone response to facilitate infection (Table 2) (El Hadrami et al., 2015).

These studies present an attractive avenue for future research, as the putative role of detoxification metabolites in actively facilitating infection has been largely overlooked. A potential example of this can be seen in the conversion of the flavanone sakuranetin into naringenin by the rice pathogen *M. oryzae* during infection (Katsumata et al., 2017). This process is assumed to be aimed at depleting the host of the antifungal sakuranetin, but naringenin is also a known inhibitor of lignin biosynthesis, revealing the possibility that its accumulation at the site of infection may suppress defensive lignification by the host (Deng et al., 2004). Moving into the future the effect of these detoxification metabolites on host physiology and defense must be evaluated as their role in pathogenesis may be as or more critical than the depletion of the initial PDC.

## Manipulating Fungal Detoxification in Disease Control

Disruption of fungal detoxification activity can have a substantial effect on pathogenicity and subversion of this activity is potentially valuable in disease control across a wide range of crops. A novel approach to disease control through the subversion of fungal detoxification has been demonstrated with the usage synthetic compounds, termed paldoxins. Paldoxins, coined from “phytoalexin detoxification inhibitors,” are rationally designed compounds which inhibit the enzymatic detoxification mechanisms used by plant pathogens without the antifungal bioactivity seen in phytoalexins. This concept was originally demonstrated with the indoles cyclobrassinin and camalexin, which competitively bind to and inhibit brassinin degrading enzymes (BH and BO discussed in section “Enzymatic Detoxification of Inducible PDCs – *Indoles*”), but as phytoalexins themselves these compounds do not qualify as paldoxins. As opposed to other fungicides which are directly toxic to the pathogen itself, paldoxins would potentially allow for the plant to defend itself from invasion without the off target ecological effects of a broad-range fungicide (Pedras and Abdoli, 2017).

Inhibitors of ABC transporter-mediated efflux have also been proposed as a method to control fungal diseases, albeit in combination with chemical fungicides. Efflux is a major mechanism of derived fungal resistance to fungicides and by applying synthetic efflux inhibitors in combination with certain fungicides, it was shown that resistant isolates can be reverted to baseline levels of susceptibility, thus improving chemical control through fungicides (Reimann and Deising, 2005).

Reduced virulence is also achieved in a number of fungal pathogens through the silencing of pathogenicity and developmental factors (McLoughlin et al., 2018; Rosa et al., 2018). The usage of host-induced or spray-induced gene silencing (HIGS or SIGS) has never directly been used on genes involved in fungal detoxification, but as many studies demonstrated the critical importance many of these genes in host colonization, they may prove to be valuable targets for RNAi approaches (Bowyer et al., 1995; Vela-corcía et al., 2019).

## Conclusion

**FIGURE 2:**
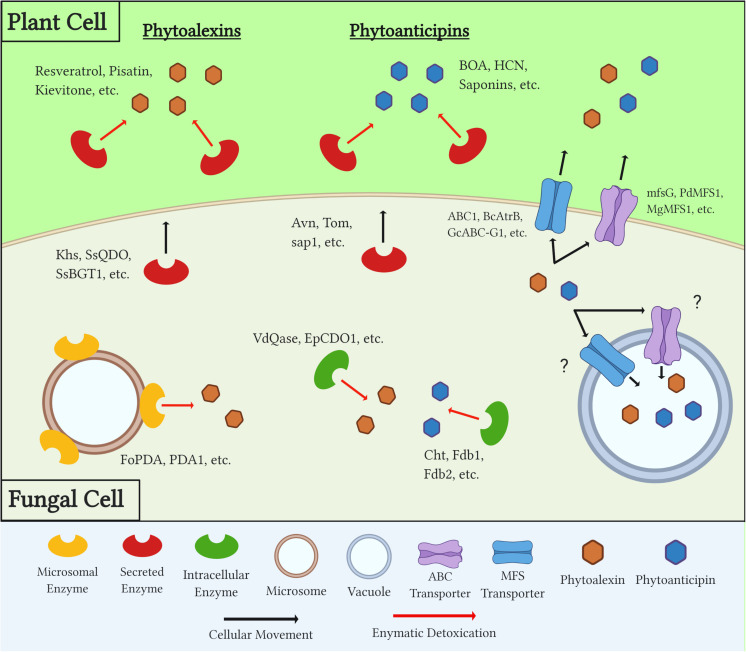
Diagram outlining generalized mechanisms of fungal tolerance for preformed and inducible plant defense compounds (PDCs). Examples of each class of PDC, detoxification enzyme, and transporter are included. Question marks denote a mechanism which is suspected but has yet to be confirmed through experimental evidence.

In order to overcome both a plant’s induced and preformed PDCs during necrotrophic colonization, fungal pathogens utilize a number of distinct mechanisms (Figure 2). The earliest response to these compounds is the production of efflux transporters that work to export, or potentially sequester, toxic compounds below a lethal and/or inhibitory threshold. Secreted enzymes are then produced and metabolize PDCs in extracellular spaces, both to protect the pathogen from these compounds and, in some cases, prevent the production of other downstream antimicrobials. Many detoxification enzymes are intracellular as well, and the localization of these proteins either inside the fungal cell or in extracellular spaces can be a factor of the protein’s mechanism of action, the chemistry of its target substrate, and/or the PDC’s target in the fungus (i.e., plasma membrane vs. mitochondria) (Figure 2 and Table 2).

These detoxification mechanisms provide several benefits to the pathogen as they can quantitatively increase virulence and in some cases expand its host range to previously resistant plants. Additionally, when efficiently metabolized the formally toxic PDCs can serve as a valuable nutrient source during colonization. Overall, the range and redundancy of these mechanisms are evidence for the importance they play in most if not all necrotrophic/hemibiotrophic infections of plants, and a better understanding of these mechanisms will undoubtedly improve disease control strategies.

## Author Contributions

NW wrote the manuscript. NW, MK, and DS revised the manuscript. All authors read and approved the manuscript.

## Conflict of Interest

The authors declare that the research was conducted in the absence of any commercial or financial relationships that could be construed as a potential conflict of interest.
